# Factors influencing the length of stay in the moroccan intensive care unit in patients surviving critical COVID-19 infection

**DOI:** 10.1016/j.amsu.2022.104108

**Published:** 2022-06-28

**Authors:** Hamza mimouni, Amine Bouchlarhem, Amine Lafkih, Leila Haddar, Oussama Lamzouri, Houssam Bkiyar, Brahim Housni

**Affiliations:** a Faculty of Medicine and Pharmacy, Mohammed I^st^ University, Oujda, Morocco; b Department of Anesthesiology and Intensive Care Unit, Mohammed VI University Hospital Mohammed I University, Oujda, Morocco; c Mohammed First University, Faculty of Medecine and Pharmacy, LAMCESM, Oujda, Morocco

**Keywords:** Covid-19, Intensive care unit, Length of hospital stay, Surviving patients, Linear regression

## Abstract

**Introduction:**

our objective is to determine the factors that influence the length of hospitalization of patients admitted to an intensive care unit.

**Methods:**

We have conducted a mono-centric retrospective cohort of 417 patients admitted in intensive care unit for a critical infection by COVID-19, for this purpose we have realized an analytical study using the linear regression model.

**Results:**

In our study, the average length of hospitalization for a critical infection with COVID-19 is 6 days (SD = 7Days), regarding the factors that influence the length of hospitalization, the length of time between the consultation and the onset of symptoms higher thann 8 days affects the length of hospitalization (coefficient = 1.2 days; CI = 0.769; 2.102 and pValue = 0.009), the presence of obesity which also affects the length of hospitalization (Coefficient = 1.6 days CI ((0.009; 3.265), and pValue = 0.049). During hospitalization, the use of mechanical ventilation, the use of tocilizumab, having a billateral nosocomial pneumonia are all factors that impact the length of hospitalization.

**Conclusion:**

It is recommended to emphasize the importance of early consultation after the onset of respiratory symptoms in the patients who are admitted to the intensive care unit in order to improve the length of their stay.

## Highlights

• Our objective is to determine the factors that influence the length of hospitalization of patients admitted to an intensive care unit.
• The average length of hospitalization for a critical infection with COVID-19 is 6 days (SD = 7Days).
• The length of time between the consultation and the onset of symptoms higher than 8 days affects the length of hospitalization.
• During hospitalization, the use of mechanical ventilation, the use of tocilizumab, having a billateral nosocomial pneumonia are all factors that impact the length of hospitalization.

## 1 Introduction

In December 2019, the emergence of cases of an atypical severe acute respiratory syndrome has been declared by China [1], the evolution was marked by the spread and the appearance of many new cases, putting the health professionals in a critical situation. In January 2020, the cause of this severe acute respiratory syndrome was finally determined, it is the infection by a virus belonging to the family of beta-coronavirus: it is the new coronavirus, and subsequently the conditions were named: the severe acute respiratory syndrome to COVID-19 (SARS-COV-2) [2]. The epidemiological situation in China worsened because of the quick appearance of many cases all across the country, as a result the infection by COVID-19 was declared in most of the countries of the world. On the first hand that there wasn't enough known scientific data about the transmission, the pathophysiology and the management of this infection, and on the other hand, that most of the health systems were not ready to manage such a global pandemic. In March 2020, the World Health Organization declared the COVID-19 infection as a global pandemic [1].

It should be pointed out that the world has experienced two outbreaks of infection by a virus of the beta-coronavirus family, namely the severe acute respiratory syndrome with COVID (SARS-COV) in November 2002 in Guangdong Province, China [3]. 26 countries reported the emergence of this virus, but the majority of cases were in China, then in 2012 in Saudi Arabia, the world experienced the emergence of new cases of infection by a virus of the beta-coronavirus family, the condition was known as the Middle East respiratory syndrome (MERS-COV) [4], 27 countries reported the emergence of MERS-COV cases, but 80% of cases were reported in Saudi Arabia. MERS-COV is characterized by more severe cases and with a very high mortality rate reaching 33% [3].

The cause of this pandemic is still a subject of research. They suppose that the virus was transmitted from bats, which represent the intermediate hosts between the virus and humans [5]. The clinical characteristics of SARS-CoV-2 resemble to the SARS-COV and Â to the MERS-COV, the incubation period varies between 5 and 15 days, when the human-to-human transmission takes place [6], the infection by the SARS-CoV-2 is transmitted by aerosols and/or droplets generated by the cough of the infected patients [7]. After transmission, COVID-19 penetrates the cell because of the affinity between the protein S which is contained in the virus wall and the angiotensin-converting enzyme-2 (ACE-2) receptor [8]. The ACE-2 receptor is mainly expressed in alveolar epithelial cells type 2 and in the respiratory epithelial cells, which explains the respiratory tropism of the virus [9]. The ACE-2 receptor is also expressed in the myocardium, ileal and esophageal epithelial cells, proximal tubular cells of the kidney and in the urothelial cells of the bladder.

In this research, we focused on the factors that impact the length of hospitalization of patients admitted to an intensive care unit with severe SARS-COV-2 infection. After the announcement of the infection as a global pandemic, many health systems claimed a great difficulty in managing the massive flow of patients even in developed countries, such as Italy [10], which made us imagine the difficulty of managing severe cases in developing countries.

The hospitalization unit of patients infected by SARS-COV-2 depends mainly on the severity of the disease, these places can be simple isolation wards [11], medical wards for moderate and mild cases not requiring ventilatory or circulatory support, and finally the intensive care units for severe and fatal cases which require mechanical ventilation or extracorporeal oxygen therapy [12].

For this purpose, we conducted a retrospective study between March 2020 and February 2021 in our intensive care unit of CHU MOHAMMED VI OUJDA to determine the factors that can impact the length of the hospitalization of patients admitted for severe SARS-COV-2 infection.

## 2 Materiels et methodes

### 2.1 Study setting

The intensive care unit of CHU MOHAMMED VI OUJDA played a major role in the management of the COVID-19 pandemic in MOROCCO. This unit has started hospitalizing patients since March 05, 2020. The initial capacity of this unit has increased from 20 resuscitation beds equipped with all the latest technologies to 95 resuscitation beds equipped with monitors, high-tech respirators, hemofiltration machines and plasma exchange and thus it is the national center of reference in circulatory assistance allowing advanced management of serious cases.

Admission to our unit is based mainly on clinical instability, all patients with RT-PCR for SARS-COV-2 or suspected infection with any of the following criteria:

• RF greater than 30 cpm
• SpO2% on room air less than 90%.
• Signs of respiratory struggles
• Hemodynamic instability
• Altered consciousness is admitted to our unit

All patients were closely monitored clinically by paramedical staff, and the biological monitoring was performed daily.

All patients have benefited from cardio-pulmonary ultrasound monitoring on a multi-daily basis. And all patients with hemodynamic instability received advanced invasive hemodynamic monitoring in our department.

### 2.2 Type and aims of the study

Our objective is to determine the factors that influence the length of hospitalization of patients admitted to our intensive care unit for severe COVID-19 infection. To accomplish our research, we conducted a single-center retrospective cohort in the intensive care unit of CHU MOHAMMED VI of OUJDA for the patients admitted between March 2020 and February 2021. All patients admitted to the unit fulfilled the criteria of the admission to the study.

### 2.3 Study participations

**Fig. 1:**
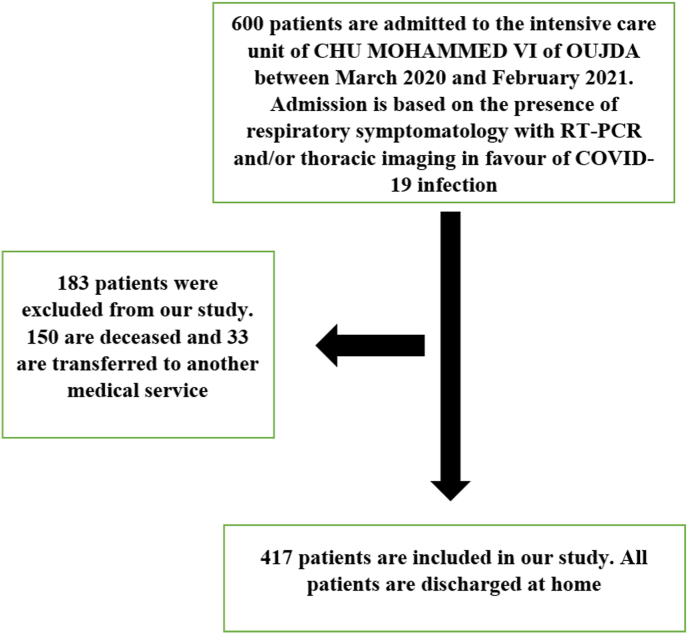
The criteria for inclusion and exclusion in the st.

The patients included in the study are: All patients admitted to the intensive care unit of CHU MOHAMMED VI OUJDA with a positive RT-PCR to COVID-19 or with a thoracic Imaging evoking the infection by COVID-19, whose evolution is the discharge at home. Patients transferred to another department for further management or who died were excluded from the study. Fig. 1.

### 2.4 Data collection

The medical information was collected between March 2020 and February 2021 by the medical staff of the unit and stored in the database of the medical observations used in the CHU MOHAMMED VI OUJDA. For each patient, the collection considered demographic information (sex, age). The medical and surgical antecedents, the anamnestic data concerning the infection including the duration of the symptoms, the duration between the beginning of the symptoms and the consultation, the symptoms of the infection by the COVID-19 and the treatment followed before the infection, the biological and imagery information and finally the treatment used during the hospitalization.

### 2.5 Statistical analysis

The objective of the study was to determine the factors that influence the length of the hospitalization of patients who were hospitalized in our intensive care unit and discharged back home.

We initially performed a descriptive analysis of the clinical, biological and therapeutic demographic characteristics of the patients. The descriptive analysis of continuous variables was expressed by a median, and categorical variables by frequency and percentage. All data were analyzed using the Statistical Package for Social Science (IBM SPSS 26).

To determine the factors influencing the length of hospitalization, a linear regression statistical analysis was applied. First, a univariate approach was performed for all the covariates, and then a multivariate analysis was performed for all the covariates that showed a significant statistical result in the univariate analysis. The associated 95% confidence interval (CI) and two-sided p-value were reported. For all statistical tests, a p value less than 0.05 was considered statistically significant.

### 2.6 Ethical approval

This study does not require a formal ethical committee approval. Access to patient data was authorized by the Mohammed VI university hospital and approved by the head of the department, taking into ac-count the retrospective design of this study. The requirement of patient consent has been lifted. Data anonymity was respected in accordance with national and international guidelines.

## 3 Results

1) Demographic, anamnestic, and medical-surgical characteristics.

In our study, 417 patients were selected. All of these patients had respiratory symptoms associated with a positive RT-PCR for COVID-19 and/or chest imaging suggestive of COVID-19 infection. The median age of our patients was 64 (SD = 18), 64% were female and 26% were male. The average duration from the beginning of the symptoms to the consultation is 7 days (SD = 5), the median hospitalization is 6 days (SD = 7). Regarding the medical history, diabetes and hypertension were the main co-morbidities with a percentage of 39.5% and 32.1% respectively. 15.1% of the patients had cardiac disease, and obesity was present in 16.7%. Table 1.

2) Clinical and paraclinical characteristics

**Table 1:** Demographic, anamnestic, and medical-surgical characteristics.

Variable	Median (±SD) or Number (%)
**Demographic carachteristics**	
Age	64 (±18)
Gender:	
- Male	267 (64%)
- Female	150 (36%)
**Anamnestic carachteristics:**	
Duration of symptoms before consultation	7(±5)
Duration of hospitalization	6(±7)
**Antecedant médicaux:**	
Diabetes	165 (39,5%)
High blood pressure	134 (32,1%)
Heart disease	63 (15,1%)
COPD	4(0,9%)
Chronic renal failure	34(8,2%)
Asthma	22(5,27%)
Active smoking	33(8%)
Obesity	70(16,7%)

COPD: Chronic obstructive pulmonary disease.

On admission to the intensive care unit, all patients underwent a complete clinical evaluation. The median BMI of our patients was 25.12 (SD = 8.47), fever was present in 291/417 or 70%, dyspnea was present in 237 patients or 56.8%. 183 patients had headaches, or 44%. The median pulse saturation on admission to room air in our patients was 88%, with a minimum at 30% and a maximum at 92%.

All patients had a complete biological workup on their admission. The median LDH was 524 (UI/L) (SD = 523), Ferritin 701(ug/l) (SD = 5445), CRP 174(mg/l) (SD = 113), D-DIMERES 1.34 (mg/l) (SD = 9) and fibrinogen 6.7(g/l) (SD = 1.9). In the blood count, median platelets at 253( × 10^3^/μl) (SD = 159), lymphocytes at 840(/ul) (SD = 2731) and White blood cells at 10 460 (/ul) (SD = 10 831). Median blood glucose at admission was 1.44 (g/l) (SD = 1.04).

Arterial blood gas on admission was performed in 398 of 417 patients, median PaCO2 was 34 mmhg (SD = 11.05), PaO2 was 60 mmhg (SD = 28.47), pH was 7.42 (SD = 0.2), and SaO2 was 88% (SD = 8.47).

For the thoracic imaging, at admission all patients had a thoracic CT scan, 376 of which were injected with contrast medium, to assess the degree of involvement. The parameters taken into account in the analysis were the degree of involvement, the appearance of the lesions and their CORADS classification, the presence or not of an intra-thoracic gas effusion, the appearance of the pericardial sac and the mediastinum, and finally the presence or not of a pulmonary embolism. 47 of the patients had an invasion between 10 and 25%, 84 patients had an invasion between 25% and 50%, 145 patients had an invasion between 50% and 75% (34.7%), and finally 141 patients had a critical invasion between 75% and 100%. Table 2.

3) Therapeutic characteristics

**Table 2:** Clinical and paraclinical characteristics.

Variable	Median (±SD) or Number (%)
**Clinical carachteristics**	
BMI	25,12 (±8,47)
Fever	291 (70%)
Dyspnea	237 (56,8%)
Asthenia	275 (66%)
Headache	183 (44%)
SpO2% on admission	88 (±8)

**Biological Findings:**	
LDH(UI/l)	542 (±523)
Ferritin(ug/l)	701 (±5445)
CRP (mg/l)	174 (±113)
D-DIMERE (mg/l)	1,34(±9)
White blood cells (/ul)	10 460(±10 831)
Lymphocytes (/ul)	840 (±2731)
Platelets ( × 10^4^/μl)	253 (±159)
Blood glucose (g/l)	1,44 (±1,04)
Fibrinogen (g/l)	6,7 (±1,9)

**Arterial blodd gaz result**	
PaCO2	34 (±11,05)
PaO2	60 (±28,47)
pH	7,42 (±0,2)
SaO2	84 (±8,47)

**Chest CT scan**	
10–25%	47 (±11,3)
25–50%	84 (±20,2)
50–75%	145 (±34,7)
+75%	141 (±33,8

LDH:Lactate dehydrogenase; CRP:C-reactiveprotein; CT: computed tomography.

From a therapeutic point of view, all our patients were put under the following national anti-COVID therapeutic protocol: Azithromycin 500 mg on the first day, then 250 mg on the following four days, Zinc 45 mg every 12 h, Vitamin C 1000 mg every 12 h, and a course of Vitamin D 100 000 IU every week. Anticoagulation was applied in 369 patients, the others were not given any treatment because of the risk of bleeding, mainly due to the presence of thrombocytopenia on admission or a lesion with a high bleeding risk. Dexamethasone was used in 123 patients (29.5%), the criteria for indication of dexamethasone were mainly an inflammatory balance sheet with a CRP higher than 15 mg/L, and finally tocilizumab: Interlokin 6 inhibitors were used in 34 of the 417 patients, or 8.1%. The use of the latter was indicated in patients with a clinical and biological exacerbation of the disease, with an Interlokin 6 level higher than 3 times normal.

Antibiotic therapy was prescribed in 167 patients, the association of Ceftriaxon with ciprofloxacin was indicated in first place, among these 167 patients 69 patients had germs with non-sensory bacteriological results, which made them switch to the triple therapy Tasocillin, Amikacin and Voriconazole.

As for oxygen therapy, oxygen goggles were used in 315 patients, especially in the weaning phase, the high concentration mask was used in 392 patients, or 94% of the patients, and the majority of the patients on admission were put on a high concentration mask, high flow oxygen therapy was used in 267 patients, non-invasive ventilation was used in 140 patients, and finally mechanical ventilation was used in 20 patients among 417. Concerning the prone position, it is used in 265 patients by sessions of 16 h per day in alternation. Table 3.

4) Evolution and complications of our patients

**Table 3:** Therapeutic characteristics.

Variable	Number of patients (%)
**Oxygenotherapy**	
Oxygen canules	315 (52,2)
High concentration mask	392 (94%)
High flow oxygen therapy	267 (64%)
Non-invasive ventilation	140 (33.5%)
Invasive ventilation	20 (4,8%)
Prone position	265 (63,5%)

**Pharmacological treatment**	
Azithromycin	417 (100%)
Vitamin C	417 (100%)
Zinc	417 (100%)
Vitamin D	417 (100%)
LMWH	369 (88,4%)
Dexamethasone	123 (29,5%)
Tocilizumab	34 (8,1%)

**Antibiotics**	
Ceftriaxone + Cifprofloxacin	167 (40%)
Tasocillin + Amikacin + Voriconazole	69 (16,5%)

LMWH: Low-molecular-weight heparin.

About the evolution of our patients, it is important to mention that all the patients were discharged at home, since this is a requirement for inclusion in our study. Thromboembolic events represent the majority of the complications with a number of 127 events between pulmonary embolism and arterial ischemia, that is to say 30,45%. In second place we find acute renal failure with a number of 103 events (24.7%), thrombocytopenia in 67 patients, pleurisy in 34 patients, pneumothorax in 8 patients and finally pleurisy in 34 patients. Table 4.

5) The factors influencing the length of the hospitalization of patients

**Table 4:** Evolution and complications of our patients.

Variable	Number (%)	%
**Thrombo-embolic events**		
Pulmonary embolism	64	15,3
Arterial ischemia	67	16,06
Both	8	1,91

**Cardiac complications**		
Myocarditis	10	2,39
Pericarditis	1	0,2

**Pleuropulmonary Complications**		
Pneumothorax	8	1,91
Pleurisy	34	8,15
Pneumomediastinum	9	2,15
Bilateral nasocomial pneumonia	67	16,06

**Biological Complications**		
Thrombocytopenia	33	7,91
Acute renal failure	103	24,7

In order to identify the factors that influence the length of hospitalization of our patients, a linear regression analysis of all the variables already presented in the descriptive analysis was performed. All variables were studied by the univariate analysis.

In univariate analysis, we found that the duration of hospitalization is influenced by: the duration of symptoms before the consultation, obesity, presence of dyspnea at admission, CRP value at admission higher than 300 (mg/l), A lymphocyte count on admission of less than 1000/μl. PaO2 below 60mmhg and SaO2 value less than 90%. in the arterial gasometry at admission, presence of a parenchymal damage higher than 50% in the thoracic CT scan, the need for invasive ventilation or mechanical ventilation during hospitalization, the use of Tocilizumab, and finally in the complications that can impact the length of hospitalization in univariable analysis are pulmonary embolism, thrombopenia, Bilateral nosocomail pneumonia (Table 5).

**Table 5:** Univariable and multivariable analysis results.

Variables	Univariable analysis			Multivariable analysis		
	Coefficient	95% CI	p. Value	Coefficient	96% CI	p. Value
**Demographic and anamnestic caracteristics**						
Age	0,016	(-0,18; 0,05)	0,362			
**Gender**						
- Male	Reference					
- Female	− 0,649	(-1,96; 0,67)	0,34			
Duration of symptoms before admission						
≤7 days	Reference					
≥8 days	3,165	**(**1,041; 5,289)	**<0.001**	1,201	(0,76; 2,102)	**0,009**
Diabtes	−0,336	(-1,673; 1,002)	0,622			
Hypertension	0,977	(-0,353; 2, 307)	0,150			
Heart disease	−0,76	(-2,593; 1,074)	0,416			
Active smoking	−0,896	(-3,3; 1,508)	0,464			
Obesity	2,36	(0,707; 4,014)	**0,005**	**1,637**	(0,009; 3,265)	**0,049**
**Clinical caracteristics**						
BMI	0,035	(-0,19; 0, 088)	0,205			
Fever	1,168	(-0,285; 2,621)	0,115			
Dyspnea	1,935	(0,578; 3,291)	**0,005**	**-**	**-**	**-**
Cough	1,338	(-0,07; 2,745)	0,062			
Asthenia	0,249	(-1,06; 1,55)	0,709			
Headache	0,675	(-0,573; 1,923)	0,289			
**Biological findings at admission**						
LDH						
≤600UI/L	Reference					
≥600UI/L	0,001	(0,00; 0,002)	0,255			
Ferritin (ug/l)						
≤500	Reference					
≥500	−2,6	(1,003; 1,012)	0,965			
CRP (mg/l)						
≤300	Reference					
≥300	0,006	(0,00; 0,011)	**0,044**	**-**	**-**	**-**
D-DIMERE (mg/l)						
≤1	Reference					
≥1	−0,023	(-0,11; 0,064)	0,602			
White blood cells(/ul)						
≤ 10^4^	Reference					
≥ 10^4^	−1,6	(-0,016; 0,019)	0,421			
Lymphocytes (/ul)						
≤4000 and ≥ 1000	Reference					
≥4000	−0,8	(-2,1; −1,04)	0,542			
≤1000	−1,7	(-3,3; −0,137)	**0,03**	**-**	**-**	**-**
Blood glucose (g/l)						
≤1,5	Reference					
≥1,5	−0,130	(-0,731; 0,474)	0,675			
Fibrinogen (g/l)						
≤4	Reference					
≥4	0,181	(-0,163; 0,524)	0,302			
**Arterial blood gas test at admission to the ambient air**						
PaCO2 (mmhg)						
≤50 and ≥ 30	Reference					
≤30	0,651	(-1,12; 1,039)	0,761			
≥50	−0,021	(-0,08; 0,039)	0,496			
PaO2 (mmhg)						
≥60	Reference					
≤60	1,349	(-0,071; 2,026)	**<0.001**	**-**	**-**	**-**
pH						
≤7,5 and ≥7,2	Reference					
≥7,5	0,78	(1,03; 2,602)	0,387			
≤7,2	1,651	(-1,601; 4,902)	0,319			
SaO2 (%)						
≥90	Reference					
≤90	1,177	(-0,255; 1,8)	**<0.001**	**-**	**-**	**-**
**Chest CT scan a admission**						
50–75%	1,67	(1,446; 2,605)	**0,013**	**-**	**-**	**-**
75–100%	2,41	(0,827; 1,182)	**<0.001**	1,321	(0,629; 1,988)	**0,017**
**Oxygenotherapie**						
High concentration mask	0,857	(-0,674; 2,387)	0,272			
High flow oxygen therapy	1,4	(0,159; 2,634)	0,127			
Non invasive ventilation	2,15	(0,695; 3,618)	**0,004**	0,068	(-0,181; 2,686)	0,087
Mechanical ventialtion	4,867	(3,6; 6,134)	**<0.001**	2,353	(0,775; 3,97)	**0,04**
Prone position	0,35	(-1,141; 4,567)	2,15			
**Traitement**						
Tociluzimab	4,6	(2,9; 6,451)	**<0.001**	3,103	(1,312; 4,894)	**0,001**
Steroides	1,059	(-0,462; 2,579)	0,172			
Antibiotics	0,17	(-0,176; 1,189)	0,367			
**Complications during hospitalization**						
Pulmonary embolism	3,632	(1,102; 6,162)	**0,005**	**-**	**-**	**-**
Arterial ischemia	−1,289	(-2,884; 0,305)	0,113			
Acute renale failure	2,151	(0,851; 3,451)	1,623	**-**	**-**	**-**
Thrombopenia	5,736	(3,474; 7,998)	**<0.001**	**-**	**-**	**-**
Bilateral nosocomial pneumonia	5,143	(3,493; 6,792)	**<0.001**	2,576	(0,458; 4,689)	**0,019**
Pneumothorax	1,031	(0,671; 1,391)	1,718	**-**	**-**	**-**

In multivariable analysis, we concluded that the factors that impact the length of hospitalization according to our cohort are: the duration of symptoms before admission more than 8 days with a duration of more than 1.2 days in patients with delayed admission (p. Value 0.009), the presence of obesity with a duration of more than 1.6 days (p. Value 0.049), the fact of having an impairment between 75 and 100% with a coefficient at 1,321 (p.Value **0,017)**. During hospitalization, the factors that had an impact were the use of mechanical ventilation with a duration of more than 2.3 days in intubated patients (p. Value 0.04, the use of tocilizumab with a duration of more than 3.1 days in patients who received it (p. Value 0.001) and finally, the billateral nosocomail pneumonia can also extend the hospitalization more than 2.5 days (p. Value 0.019) (Table 5).

## 4 Discussion

In our retrospectively collected cohort, the median age was 64 years with SD of 18, the majority of patients were male with a proportion of 64%, the same result was reported by Grasseli et al. concerning the median age of 63 years but with a higher proportion of males reaching 82% [13]. According to a systematic review on the clinical characteristics of patients admitted to the ICU for critical infection with COVID-19, the mean age was 56 years, and always with a higher proportion of men [14]. The duration of evolution of the symptomatology before the consultation and the beginning of the national anti-covid protocol was 7 days with SD to 5 days, Wu et al., reported a median of 13 days, but in patients admitted in a medical service for a moderate form of the disease [15]. Regarding comorbidities, diabetes was present in 39% of the patients, currently it is proven that diabetes should be considered as a risk factor of a critical evolution of the disease [16]. Secondly, arterial hypertension in China, 26% of infected patients are hypertensive [17], with an unfavorable evolution whenever this hypertension is not well controlled. The presence of pre-existing heart disease is a prognostic factor of the disease, so the clinician will be faced with the challenge of managing the severe infection on the one hand, and on the other hand the heart disease that in the majority of cases is decompensated [18].

Regarding the clinical features, fever is the most frequent symptom, even reported in a meta-analysis by Alfonso et al. [19], the biological exploration objectified very disturbed inflammatory markers, it is the expression of the cytokinic storm secondary to the immune reaction of the host [19]. Ferritin, CRP, LDH, and D-DIMERE were the main inflammatory markers measured. Interlukine-6 was measured if there was a suspicion of release cytokine syndrome secondary to COVID-19 [20].

Arterial gasometry was performed to determine the degree of hypoxemia, and thus to classify the severity of the acute respiratory distress syndrome by calculating the PaO2/FiO2 ratio [21]. The most observed complications were venous and arterial thrombo-embolic events. It should be noted that thromboembolic events are a real challenge to manage during COVID-19 infection. In our study the percentage of thromboembolic events is about 30.45%, the same results reported by Akta et al. [22]. The mechanism of these accidents is multifactorial ranging from microangiopathy secondary to infection to hypercoagubility explained by the important inflammatory syndrome [23]. Another mechanism that may explain these events is new-onset atrial fibrillation secondary to infection or the combination of diabetes and hypertension in an infected patient [24]. Acute renal failure was observed in 24.7%, the same results reported by Gabarre and al with a result of 25%. Among the 417 patients included, the infection was revealed by pericarditis [25], 10 others were complicated by myocarditis. Regarding the superinfection of pulmonary lesions, it is observed in 16% of patients, while Musuuza and al reported the percentage of 24% in a meta-analysis regarding the prevalence and evolution of superinfections in patients infected with COVID-19 [26], with an unfavorable evolution and a higher mortality.

About the purpose of our study, the median length of hospitalization is 7 with a SD of 5, the same length is reported in a systematic review concerning the length of hospitalization in patients infected with SARS-COV-2 [12].

Our median mainly involved the length of hospitalization of the patients whose evolution was the discharge at home, according to a cohort of 1591 Patients Infected with the SARS-CoV-2 admitted to the ICUs of the Lombardy Region, Italy, 8 days is the median of the discharged patients, one more day in our ICU [13], a cohort of 2249 patients hospitalized in the ICU for a critical COVID-19 infection with the results of the discharge at home showed a median of 5 days [12]. In the other hand, in China the median was a little bit higher, it varies between 8 and 13 days as it was shown by Cao and al [27].

A Regarding the factors that may influence the length of hospitalization, it has been found that the length of time between the onset of symptoms and the start of the anti-covid protocol prescribed in our country influences the length of hospitalization. Wu and al reported that a short time between the onset of symptoms and hospital admission is associated with a higher length of the hospitalization, but their study was based on patients with mild or moderate disease [15], Obesity is also associated with a higher length of hospitalization, and this can be due to the impact of the obesity on the respiratory physiology, the immunity and thus the comorbidities [28], if the damage is higher than 75% it increases the duration of hospitalization, and this is explained by the difficulty of a fast respiratory weaning considering the degree of damage.

Regarding the intra-hospital factors that influenced the length of hospitalization, the use of mechanical ventilation and the use of tocilizumab prolonged the duration of use, and this can be explained on the one hand by the infections acquired under mechanical ventilation, and on the other hand by the state of immunosuppression generated by tocilizumab, which represents a risk factor for developing a secondary infection, which may prolong the duration of hospitalization [26]. The use of the prone position could decrease the length of hospital stay and this can be explained by the fact that it improves gas exchange, the improvement of the PaO2/FiO2 ratio and the decrease of intra-pulmonary shunts [29].

Concerning the complications that influence the length of hospitalization, we find nosocomial pneumopathies, a classic factor that affects the length of hospitalization of the patients admitted in ICU, the same results is reported by Wu and al [15], and finally pneumothorax, also affected the length of hospitalization.

## 5 The limitations

Our results are represented by the fact that we conducted a mono-centric study with a small number of patients, as well as the determination of the duration between the onset of symptoms and the consultation is specified by the patient himself, which may reduce the duration due to the subjective interpretation of symptoms.

This case series has been reported in line with the STROCSS guidelines [30].

## 6 Conclusion

Our study demonstrates that the delay of medical consultation when the patient has respiratory symptoms and the obesity are the main patient-dependent factors influencing the length of ICU stay after severe COVID-19 infection in survivors.

## Ethical approval

The ethical committee approval was not required give the article type.However, the written consent to publish the clinical data of the patients was given and is available to check by the handling editor if needed.

## Sources of funding

None.

## Author contribution

HAMZA MIMOUNI: study concept or design, data collection, data analysis or interpretation, writing the paper. AMINE BOUCHLARHEM: study concept or design, data collection, data analysis or interpretation, writing the paper. AMINE LAFKIH: data collection. LEILA HADDAR: data collection, data analysis, study concept. OUSSAMA LAMZOURI: data collection. Houssam bkiyar: supervision and data validation. Brahim Housni: supervision and data validation.

## Trial registry number

Researchregistry7892.

## Guarantor

Amine Bouchlarhem.

## Consent

This is a retrospective cohorte study.

## Declaration of competing interest

None.
